# Reduced automaticity in freezing of gait is associated with elevated cortico-cerebellar connectivity

**DOI:** 10.1007/s11682-025-00996-w

**Published:** 2025-03-18

**Authors:** Daniel H. Lench, Aaron Embry, Niloufar Malakouti, Nathan DeTurk, Gonzalo J. Revuelta

**Affiliations:** 1 Department of Neurology, Medical University of South Carolina, Charleston, SC USA; 2 Department of Rehabilitation Science, Tufts University, Boston, MA USA; 3 Department of Health Sciences and Research, Medical University of South Carolina, Charleston SC, USA

**Keywords:** Parkinson’s Disease, Freezing of Gait, Cerebellum, Automaticity

## Abstract

Freezing of gait (FOG) in individuals with Parkinson’s Disease is associated with a loss of gait automaticity. This loss of automaticity is demonstrated by worsening gait performance while dual tasking. Functional connectivity between the cerebellar vermis and cortex have previously been associated with spatiotemporal measures of gait in PD. The objective of this study was to determine whether this corticocerebellar connectivity is associated with gait automaticity as measured by dual task interference in PD FOG. 55 participants with PD were recruited (38 FOG, 17 non-FOG controls) to undergo a resting-state functional magnetic resonance imaging scan. Gait automaticity was quantified using spatiotemporal metrics from single and dual task time up and go trials. FOG participants demonstrated shorter step length and gait velocity compared to non-FOG PD controls. A trend toward greater dual task interference of step length in the FOG group was found. Using a seed-based connectivity approach we observed that FOG participants have greater vermis connectivity than non-FOG PD participants to several cortical regions including the superior parietal lobe, supplemental motor area, precentral gyrus and posterior cingulate (voxel threshold *p* < 0.01, cluster FWE corrected *p *< 0.05). Meanwhile, vermis connectivity to the occipital cortex was reduced in FOG participants relative to non-FOG controls. Dual task interference of step length among the FOG group correlated with the degree of vermis connectivity to the sensorimotor cortex and superior parietal cortex (voxel threshold *p* < 0.01, cluster FWE corrected *p* < 0.05). We conclude that increased corticocerebellar connectivity may be associated with loss of gait automaticity in individuals with PD FOG.

**Supplementary Information:**

The online version contains supplementary material available at 10.1007/s11682-025-00996-w.

## Introduction

Freezing of gait (FOG) is a disabling feature of Parkinson’s Disease (PD) which has been hypothesized to result from a loss of gait automaticity (Hallett 2008; Vandenbossche et al., 2012). Although loss of automatic gait appears to worsen with the progressive depletion of nigrostriatal dopamine, the precise adaptations of brain circuitry in response to this loss of automaticity is unclear (Hallett 2008; Hirata et al., 2020; Vandenbossche et al., 2012). Gait automaticity can be defined as the ability to successfully execute gait in the absence of executive control and attention (Clark, 2015). Dual tasking protocols which intentionally increase cognitive and attentional resources (e.g., performing math problems, repeating the alphabet backwards) is a common approach to assess gait automaticity (Penko et al., 2018). In participants with FOG, performing concurrent cognitive or motor tasks while walking can trigger episodes of freezing (Spildooren et al., 2010). One explanation for this finding is that FOG patients require additional attentional resources to successfully maintain their gait (Spildooren et al., 2010). When there is a reduction in executive function, patients are no longer able to compensate effectively, and FOG arises (Belluscio et al., 2019). The extent to which cognitive demand interferes with automatic gait is known as dual task interference and is quantified as the relative performance of gait during a single versus a dual task. Multiple studies have reported increased dual task interference in patients with FOG (Souza Fortaleza et al., 2017; Spildooren et al., 2010).

The neural adaptations to this loss of automaticity are an area of active investigation as it has the potential to inform the pathophysiology of FOG and potential targets for therapeutic intervention. Changes in cortical-cortical and cortical-brainstem connectivity have been implicated in disrupted dual tasking behavior, however their relationship to objective measures of gait automaticity are only sparsely reported in small samples (Peterson et al., 2015). Increasing evidence implicates a role of the cerebellum and its functional connectivity in the development of FOG behavior (Fasano et al., 2017). To date, however, the contribution of the cerebellum to gait automaticity in individuals with PD remains unclear. It is widely accepted that the cerebellum integrates afferent proprioceptive information and efferent motor commands from the spinal cord, brainstem and cerebral cortex which contribute to balance and locomotion (Morton & Bastian, 2004). Early studies on control of locomotion performed in cats in which electrical or chemical stimulation were used to initiate or modulate stepping behavior, included the identification of a cerebellar locomotor region (CLR) (Armstrong, 1988). Meanwhile, the vermis of the cerebellum appears to modulate gait and interacts with the cerebral cortex (Maiti et al., 2021; O'Reilly et al., 2010). Interestingly rhythmic activity of the mesencephalic locomotor region (MLR) cells originate from the vermis and paravermal cerebellar cortex (Armstrong, 1988). Several studies in humans have used virtual reality walking and active stepping tasks in the MRI scanner demonstrate that the vermis of the cerebellum is reliably engaged (Doolittle et al., 2020; Marchal et al., 2019).

In this study we hypothesize that adaptations to the disrupted loss of automaticity of gait in FOG may result from in changes in the cerebellum’s interaction with cortical structures. Preclinical animal models have implicated cortico-cerebellar loops in motor planning behaviors (Zhu et al., 2023). In non-human primates, the vermis of the cerebellum has direct connectivity with the cortex in addition to vestibulocerebellar and proprioceptive spinocerebellar afferents (Coffman et al., 2011). Meanwhile in human studies, the cerebellar vermis has functional connectivity to sensorimotor cortical regions (Maiti et al., 2021). Connectivity of this portion of the cerebellum with cortex is associated with walking capacity in healthy adults (Boyne et al., 2018). One study demonstrated vermis connectivity to sensorimotor and supplementary cortex are altered in PD participants and a subsection of vermis correlates with spatiotemporal measures of gait performance (Maiti et al., 2021). Vermal-cortical connectivity has not however, been investigated in the context for FOG and automaticity of gait. In the current study we investigate the relationship between gait automaticity as measured by dual task interference and vermal-cortical connectivity using fMRI in a group of participants with PD FOG.

## Methods

### Participants

A total of 55 participants with Parkinson’s Disease were enrolled in an observational, cross-sectional neuroimaging study at the Medical University of South Carolina’s (MUSC) Murray Center for Research on Parkinson’s and Related Disorders. Participants were recruited from the movement disorders clinic at MUSC and the Ralph H Johnson VA hospital. This study was approved by the MUSC’s Institutional Review Board (IRB) (Pro00037836) and all participants provided written informed consent to study procedures. Inclusion criteria included a diagnosis of PD as defined by the UK Brain Bank Criteria (Hughes et al., 1992) either with or without the presence of FOG. Exclusion criteria included MRI contraindications, cognitive dysfunction (< 26 on the Mini-Mental State Examination), and inability to walk along a 30-foot walkway while in the off-state (Lench et al., 2020, 2021). Assignment to one of two groups (PD FOG versus PD non-FOG controls) was determined by whether the participant reported freezing on the new FOG Questionnaire (nFOGQ) (Nieuwboer et al., 2009) or a score ≥ 1 on Item-14 of the Unified Parkinson's Disease Rating Scale (UPDRS). Accuracy of FOG status was confirmed by the study movement disorders neurologist (GJR) based on clinical observation of walking. Concordance between subjective and clinician-based observation of FOG was required to confirm the presence of FOG and assignment to the PD FOG group. 38 participants were assigned to the PD FOG group, and 17 participants were assigned to the PD non-FOG control group.

### Gait assessment and spatiotemporal measures

Objective gait assessments were administered by a research physical therapist (AE) and were performed in the on and off medication states using a GAITRite© digital walkway (CIR Systems, Franklin NY). Since subjects in the PD non-FOG group experienced no (or minimal) motor fluctuations clinically, evaluations were only performed in the on-medication state for this group. Off-state assessments were made at least 12 hours off all dopaminergic medication (24 hours off extended-release formulations), on-state assessments were made at least 30 min after taking the first dose of medications on the same day. Spatiotemporal parameters were extracted from timed up and go (TUG) trials which were performed while either single tasking (walking at the participant’s self-selected speed) or dual tasking (serial 7 s and/or every other letter of the alphabet). Each TUG task (single and dual) was performed twice, and trials were averaged. Although FOG is known to increase during dual tasking and turning, no procedures were specifically designed to provoke FOG in this protocol. If a participant required physical assistance to prevent a fall, the trial was terminated and attempted again. Incomplete trials were not included in the final analysis. Data was extracted using GaitRite software and only complete steps were included in the final analysis of spatiotemporal parameters. In addition, all walking passes were manually checked for quality and accuracy of step identification. Steps were included in the analysis if there was clearly identifiable heel contact through the toe off 'stance phase' of gait and omitted in instances where the individual would shuffle and never clear the foot (i.e. entered the swing phase). Spatiotemporal measures included the total time for task completion, gait velocity, step length, standard deviation (SD) of step length, and cadence. Velocity was defined as the distance walked divided by the time to walk. Cadence was defined as the number of steps per minute. Step length was calculated as the anterior–posterior distance from the heel contact point of one footprint to the heel contact point of the opposite footprint. Step length coefficient of variability was calculated from step length SD values using methods previously described (Hausdorff et al., 1998). To measure gait automaticity, dual task interference measures were calculated from dual and single TUG spatiotemporal metrics (Souza Fortaleza et al., 2017). Dual task interference was calculated using the following formula:

$$\text{Dual Task Interference }= \frac{Dual \;Task \;Variable \;- \;Single \;Task \;Variable \;}{Single \;Task \;Variable} {\times}\;100$$

### Image acquisition

Anatomical and resting-state functional MRI (rs-fMRI) scanning was performed using 3 T Siemens Trio scanner at MUSC's Center for Biomedical Imaging. All scans were acquired using a 32-channel head coil. High-resolution T1-weighted anatomical scans (TR = 2300 ms, TE = 2.26 ms, TI = 900 ms, slice thickness = 1 mm, field of view = 256 mm, flip angle = 8°) and rs-fMRI scans (T2*; TR = 2200 ms, 36 transverse slices, slice thickness = 3.0 mm, interleaved or ascending slice order, 119 vol, 4 min and 30 s) were acquired in the medication on-state while participants fixated on a cross.

### RS-fMRI preprocessing

Functional and anatomical data were converted to 4D NifTI images using dcm2niix and were preprocessed using a flexible preprocessing pipeline specified in the open-source CONN toolbox (Version 19. c) as implemented in Matlab (Mathworks R2020b). Functional and anatomical data were preprocessed using a flexible preprocessing pipeline (Ashburner, 2007; Whitfield-Gabrieli & Nieto-Castanon, 2012) including realignment with correction of susceptibility distortion interactions, slice timing correction, outlier detection, direct segmentation and MNI-space normalization, and smoothing. Functional data were realigned using SPM realign & unwarp procedure (Andersson et al., 2001), where all scans were coregistered to a reference image (first scan of the first session) using a least squares approach and a 6 parameter (rigid body) transformation, and resampled using b-spline interpolation to correct for motion and magnetic susceptibility interactions. Temporal misalignment between different slices of the functional data was corrected following SPM slice-timing correction (STC) procedure (Sladky et al., 2011), using sinc temporal interpolation to resample each slice BOLD timeseries to a common mid-acquisition time. Potential outlier scans were identified using ART as acquisitions with framewise displacement above 0.9 mm or global BOLD signal changes above 5 standard deviations (Power et al., 2014), and a reference BOLD image was computed for each subject by averaging all scans excluding outliers. Functional and anatomical data were normalized into standard MNI space, segmented into grey matter, white matter, and CSF tissue classes, and resampled to 2 mm isotropic voxels following a direct normalization procedure (Calhoun et al., 2017) using SPM unified segmentation and normalization algorithm (Ashburner, 2007; Ashburner & Friston, 2005) with the default IXI-549 tissue probability map template. Last, functional data were smoothed using spatial convolution with a Gaussian kernel of 8 mm full width half maximum (FWHM). In addition, functional data were denoised using a standard denoising pipeline including the regression of potential confounding effects characterized by white matter timeseries, CSF timeseries, motion parameters and their first order derivatives (Friston et al., 1996), outlier scans (Fasano et al., 2017), and linear trends within each functional run, followed by bandpass frequency filtering of the BOLD timeseries (Hallquist et al., 2013) between 0.008 Hz and 0.09 Hz. CompCor (Behzadi et al., 2007; Chai et al., 2012) noise components within white matter and CSF were estimated by computing the average BOLD signal as well as the largest principal components orthogonal to the BOLD average, motion parameters, and outlier scans within each subject's eroded segmentation masks.

### Statistical analyses

#### Disease severity and demographics

Differences in disease severity and demographic characteristics between PD non-FOG and PD FOG groups were evaluated using 2-sample t-tests or Chi-squared tests where appropriate. Analyses were performed using SPSS Statistics (V28) software. Variables analyzed included disease duration, Hoehn and Yahr staging, levodopa equivalent daily dose (LEDD), mini mental state examination (MMSE), UPDRS part-III scores. Group differences were considered significant if *p* < 0.05.

#### Spatiotemporal and dual task interference measures

To assess differences in gait spatiotemporal and dual task interference measures, 2-sample t-tests were performed comparing the PD FOG and PD non-FOG groups. To account for the number of 2-sample t-tests performed for spatiotemporal measures (10 total) Bonferroni correction for multiple comparisons was performed. Thus, only *p* < 0.005 was considered significant. To limit the number of dual task interference measures tested, only spatiotemporal variables that differed significantly between the FOG and non-FOG groups were selected to calculate dual task interference. Comparison of PD FOG and PD non-FOG dual task interference was performed using 2-sample t-tests and considered significant if *p* < 0.05.

#### Resting-state functional connectivity

Using the vermis (IV/V) subregion as a seed, we compared connectivity between PD FOG and PD non-FOG participant groups. The vermis (IV/V) subregion was defined by CONN’s default AAL cerebellum atlas (see Supplemental Fig. 1) and was selected based off previous reports that connectivity of this portion of vermis is associated with gait impairment in PD (Maiti et al., 2021). In a follow-up analysis the association between vermis (IV/V) connectivity and dual task interference performance (for variables identified to be different between PD FOG and PD non-FOG) was assessed using a linear regression. All group-level analyses were performed using a General Linear Model (GLM) in CONN (Whitfield-Gabrieli & Nieto-Castanon, 2012). Age, disease duration and LEDD were included in the GLM models as covariates. Voxel-level hypotheses were evaluated using multivariate parametric statistics with random-effects across subjects and sample covariance estimation across multiple measurements. Inferences were performed at the level of individual clusters (groups of contiguous voxels). Results were thresholded using a combination of a cluster-forming *p* < 0.01 voxel-level threshold, and a familywise corrected p-FDR < 0.05 cluster-size threshold (Chumbley et al., 2010).

**Fig. 1:**
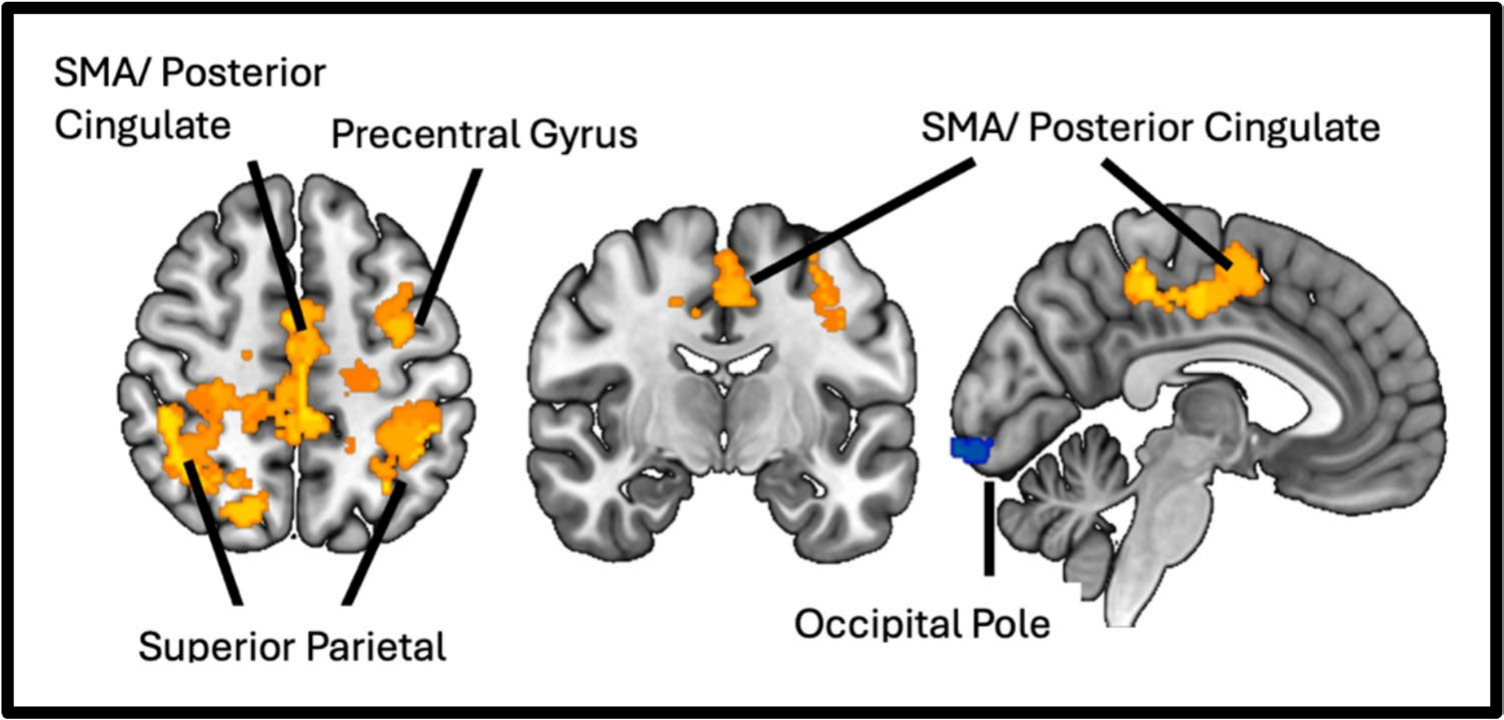
Vermis 4/5 functional connectivity in FOG vs PD non-FOG groups: Cortical areas with greater connectivity to vermis 4/5 in FOG (*n* = 38) than PD non-FOG (*n* = 17) (voxel-wise threshold *p* < 0.01, FDR corrected cluster threshold *p* < 0.05) are shown in red-yellow clusters. Cortical areas with reduced connectivity to vermis 4/5 in FOG relative to PD non-FOG are shown in blue clusters. All GLM models were corrected for age, disease duration and LEDD

## Results

### Demographics and disease severity in FOG versus non-FOG PD groups

Participant demographics and disease severity scores for the PD FOG and PD non-FOG groups can be seen in Table 1. Participants in the FOG and PD non-FOG groups did not differ in mean age, MMSE cognition scores, disease severity as measured by UPDRS Part III and H&Y staging. While there was a trend toward greater disease duration in the PD FOG group (which was expected given FOG occurs later in the disease process), this difference did not reach significance (*p* = 0.058). The control group had more years of education (*p* = 0.002) and lower levodopa-equivalent daily dose (LEDD) (*p* < 0.001) as expected.

**Table 1:** Demographics and disease severity

Participant Demographics and Disease Severity	PD with FOG (*n* = 38)	PD Controls (*n* = 17)	*P*-value
Age (years)	67.1 (7.1) years	68.6 (5.3) years	0.440
Sex (M,F)	27,11 (71%M,29%F)	14,3 (82%M,18%F)	0.374
Education (years)	15.0 (2.9) years	17.7 (2.8) years	**0.002**
Disease Duration (years)	7.8 (5.0) years	5.2 (3.5) years	0.058
H&Y Staging ON (stage)	2.1 (0.3) a.u	2.0 (0.4) a.u	0.309
nFOG-Q (score)	20.4 (3.9) a.u	-	-
UPDRS Part-III ON (score)	18.2 (6.5) a.u	18.6 (7.5) a.u	0.841
UPDRS Part-III OFF (score)	25.8 (7.6) a.u	-	-
MMSE (score)	28.5 (1.5) a.u	28.8 (1.3) a.u	0.4791
LEDD (mg)	1181.9 (586.4) mg	510 (344.6) mg	** < 0.001**

Table Abbreviations: Values presented as mean (SD). *p*-values in bold reached threshold of significance *p* < 0.05. *M* male, *F* female, *a.u.* arbitrary units, *LEDD* levodopa equivalent daily dose, *MMSE* mini-mental state examination, *UPDRS* Unified Parkinson’s Disease Rating Scale, *H&Y* Hoehn and Yahr scale

### Spatiotemporal gait measures and dual task interference

Group differences in spatiotemporal measures between PD FOG and PD non-FOG participants are displayed in Table 2. PD FOG participants had shorter steps (mean difference of 10.6 cm, *p* = 0.006) and walked at a slower velocity (mean difference of 21 m/sec, *p* = 0.004) than PD non-FOG participants during single task walking when ON dopaminergic medication. Similarly, during dual task walking on dopaminergic medication PD FOG participants had shorter steps (mean difference of 12.1 cm, *p* = 0.002) and walked at a slower velocity (mean difference of 23 m/sec, *p* = 0.003) than PD non-FOG participants. Group differences in these spatiotemporal measures were considered significant if *p* < 0.005 to correct for multiple comparisons. While single task step length did not reach this threshold, it approached significance (*p* = 0.006). Mean cadence, step length CV and time to complete the task was not significantly different between groups on dopaminergic medication. A trend toward significantly greater on-state step-length dual task interference was found in the FOG group (*p* = 0.053). No group difference in on-state velocity dual task interference were found (*p* = 0.227). A more negative step length dual task interference value represents shorter steps during the dual task relative to the single task. A more negative velocity dual task interference value represents slower gait during the dual task relative to the single task. Group differences in step length and velocity dual task interference are displayed in Table 3.

**Table 2:** Spatiotemporal Measures

Spatiotemporal Gait Measures		PD with FOG		PD Controls	*p*-value
Time to complete (seconds)					
Single Task TUG time ON	*n* = 34	29.4 (17.8) sec	*n* = 17	20.8 (4.2) sec	0.056
Single Task TUG time OFF	*n* = 34	64.8 (133.5) sec			
Dual Task TUG time ON	*n* = 34	60.1 (118.0) sec	*n* = 17	23.9 (4.7) sec	0.214
Dual Task TUG time OFF	*n *= 33	87.2 (121.3) sec			
Velocity (meters/second)					
Single Task velocity ON	*n* = 33	105.8 (22.7) m/sec	*n* = 16	126.8 (22.2) m/sec	0.004*
Single Task velocity OFF	*n* = 32	89.6 (31.7) m/sec			
Dual Task velocity ON	*n* = 33	87.1 (24.6) m/sec	*n* = 16	110.1 (22.4) m/sec	0.003*
Dual Task velocity OFF	*n* = 30	71.7 (24.6) m/sec			
Cadence					
Single Task cadence ON	*n* = 33	113.8 (11.2)	*n* = 16	114.3 (6.0)	0.857
Single Task cadence OFF	*n* = 32	109.4 (17.5)			
Dual Task cadence ON	*n* = 33	107.4 (18.6)	*n* = 16	108.9 (9.3)	0.761
Dual Task cadence OFF	*n* = 30	108.2 (18.1)			
Step Length (cm)					
Single Task step length ON	*n* = 33	56.1 (11.8) cm	*n* = 16	66.7 (12.4) cm	**0.006**
Single Task step length OFF	*n* = 32	48.1 (15.2) cm			
Dual Task step length ON	*n* = 33	48.6 (12.1) cm	*n* = 16	60.7 (11.3) cm	0.002*
Dual Task step length OFF	*n* = 30	40.1(12.8) cm			
Step Length CV					
Single Task step length CV ON	*n* = 32	7.7 (11.0)	*n* = 14	4.3 (1.7)	0.252
Single Task step length CV OFF	*n* = 31	12.5 (19.4)			
Dual Task step length CV ON	*n* = 32	10.5 (15.6)	*n* = 14	4.8 (1.7)	0.184
Dual Task step length CV OFF	*n* = 30	12.6 (11.8)			

Table Abbreviations: Values presented as mean (SD). Uncorrected *p*-values < 0.05 are in bold, *p*-values Bonferroni corrected for the number of spatiotemporal comparisons performed (10) are considered significant if *p* < 0.005 and denoted by *; *TUG* time up and go, *CV* Coefficient of variability

**Table 3:** Dual Task Interference Measures

Dual Task Interference		PD with FOG		PD Controls	*p*-value
Velocity Interference ON	*n* = 33	−18.2 (15.8)	*n* = 16	−13.0(8.1)	0.227
Velocity Interference OFF	*n* = 30	−23.5 (14.8)			
Step Length Interference ON	*n* = 33	−14.1 (10.2)	*n* = 16	−8.6 (5.4)	*0.053*
Step Length Interference OFF	*n* = 30	−21.1(13.3)			

### Functional connectivity of cerebellar vermis

**Fig. 2:**
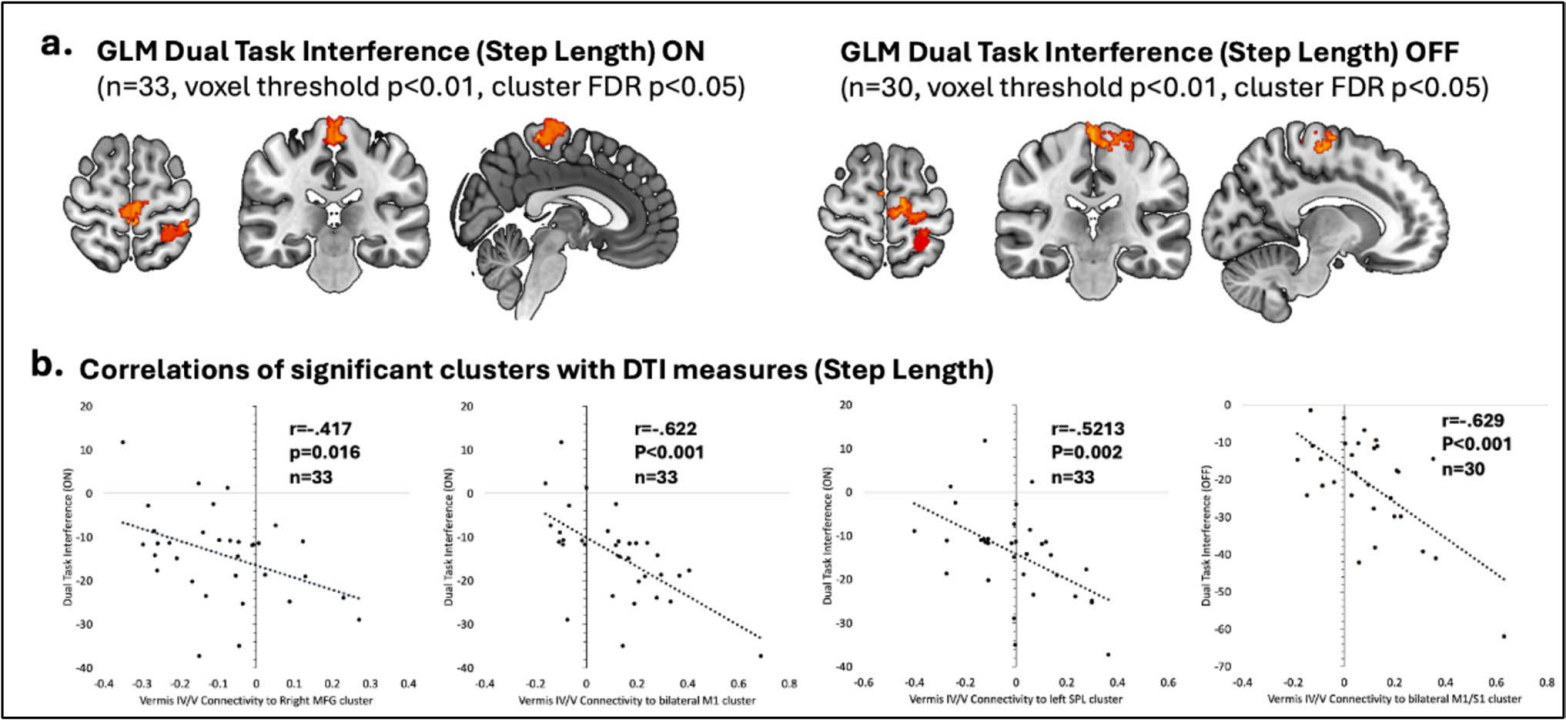
Vermis 4/5 functional connectivity associated with Dual Task Interference (Step Length): (**a**) Cortical areas associated with greater dual task interference of step length (red-yellow) using a GLM (voxel-wise threshold *p* < 0.01, FDR corrected cluster threshold *p* < 0.05) correcting for age, disease duration and LEDD. (**b**) Correlation between DTI and vermis connectivity to cortical clusters identified

Group differences in functional connectivity of the cerebellar vermis (IV/V) are shown in Table 4 and visually shown in Fig. 1. Vermis connectivity was elevated in FOG participants relative to non-FOG participants with several cortical brain regions including the left and right superior parietal lobule (SPL), postcentral gyrus, supplemental motor area (SMA), posterior cingulate cortex (PCC) and left middle frontal gyrus (MFG). Meanwhile vermis connectivity was lower in FOG participants relative to non-FOG participants with the left and right occipital pole. A GLM model was used to identify brain regions wherein functional connectivity of vermis was associated with step length dual task interference performance. Greater interference ON dopaminergic medication was associated with increased vermis connectivity to the right MFG, the left and right (medial portion) of the precentral gyrus and the left SPL. Greater interference OFF dopaminergic medication was similarly associated with increased vermis connectivity to the sensorimotor cortex and the left SPL. These findings are displayed in Table 4 and Fig. 2. All brain regions were identified using a voxel level threshold of *p* < 0.01 and clusters size threshold of FDR-*p* < 0.05.

**Table 4:** Significant Clusters from Vermis Seed-based Connectivity Analysis

	Region	MNI Peak Coordinates (x,y,z)	Cluster size	size p-FDR corrected
PD FOG > PD Non-FOG	Right Superior Parietal Lobule, Postcentral Gyrus	+ 30 −30 + 50	765	0.000005
	Left Superior Parietal Lobule, Postcentral gyrus	−44 −36 + 46	514	0.000081
	Supplemental Motor Area, Posterior Cingulate	−4 −2 + 54	512	0.000081
	Left Middle Frontal Gyrus	−44 + 6 + 38	391	0.000612
PD Non-FOG > PD FOG	Right Occipital Pole	+ 26 −84 −14	230	0.015299
	Left Occipital Pole	−18 −88 −12	580	0.00005
Dual Task Interference ON (Step Length)	Right Middle Frontal Gyrus	+ 50 + 10 + 38	349	0.000010
	Left and Right Precentral Gyrus	+ 00 −24 + 76	277	0.004262
	Left Superior Parietal Lobule	−26 −48 + 64	202	0.018481
Dual Task Interference OFF (Step Length)	Left Pre and Postcentral Gyrus, Left Superior Parietal Lobule	−26 −46 + 64	623	0.000005

## Discussion

In this observational study we sought to determine whether corticocerebellar connectivity was associated with gait automaticity in PD participants with FOG. We began by evaluating objective measures of gait automaticity between FOG and PD controls. We evaluated dual task interference of step length and walking velocity and found a trend toward increased dual task interference of step length in FOG participants. Next, we established differences in cerebellar vermis connectivity between PD FOG and PD non-FOG participants. We found increased functional connectivity of the cerebellar vermis (IV/V) in FOG participants to several cortical brain regions including the SPL, the precentral cortex, the SMA, and the PCC, and reduced connectivity to the bilateral occipital poles. Next, we determined the relationship between increased corticocerebellar connectivity and increased dual task interference. In participants with FOG, increased vermis connectivity with the sensorimotor cortex, SPL and MFG was associated with greater dual task interference. Overall, these results support the hypothesis that corticocerebellar connectivity plays a role in gait automaticity in PD FOG.

The observation of elevated corticocerebellar connectivity in FOG participants extends previous work on changes in resting state connectivity associated with FOG from our own group and others (Fling et al., 2014; Lench et al., 2020, 2021). While previous studies focusing on changes in cerebellar connectivity in FOG have primarily focused on the cerebellar locomotor region (CLR), this region is challenging to perform functional imaging studies from due to its size and adjacency to white matter (Fling et al., 2014; Mori et al., 1999). We focused on the cerebellar vermis, which is anatomically connected to automatic locomotor centers, consistently activated in fMRI gait tasks and is associated with spatiotemporal measures of gait in PD (Coffman et al., 2011; Doolittle et al., 2020; Maiti et al., 2021). Although vermis of the cerebellum is regularly recognized for its role in gait ataxia and tandem gait this region has rarely been described in the context of PD gait distrubances (Nakagawa et al., 1994). Indeed, the cerebellar vermis is involved in more than truncal stability and is integrated into networks responsible for cognition and locomotion (Festini et al., 2015; Fujita et al., 2020). This study is the first to find a relationship between increased corticocerebellar connectivity and gait automaticity in FOG. Increases in vermal connectivity could represent an adaptation to reduced automaticity although this needs to be confirmed by causal studies. The suggestion that cerebellum has a compensatory role in PD gait has been described in previous studies. For example, dual tasking during an anti-phase foot movement task increased cerebellar activation in PD participants with posture and gait impairment relative to healthy controls (Gardoni et al., 2023). In as second study PD participants had elevated cerebellar connectivity to motor and premotor cortex relative to controls (Vervoort et al., 2016). Since elevated connectivity with parietal and prefrontal cortex was associated with reduced automaticity, our findings suggest this may be a maladaptive compensatory response.

Aside from increases in connectivity, reduced vermis connectivity to the left and right occipital poles was observed in FOG participants. This extends previous studies demonstrating changes in connectivity between the visual cortex and cerebellum in PD relative to non-PD controls (Jung et al., 2020). This may be reflective of increased demand to attend to visual cues. Although the precise role of the vermis in visual processing is unclear, the region is integrated into a functional network responsible for information processing speed (Silva et al., 2019) and has been shown to negatively correlate with navigation time (Ramanoel et al., 2020).

From a behavioral perspective, we observed that FOG participants take shorter steps and walk slower than PD controls which is widely accepted and is consistently reported (Souza Fortaleza et al., 2017). This was particularly evident during dual tasking where group differences in both velocity and step-length maintained significance after correcting for multiple comparisons. However, unlike previous studies which have found significant differences in dual task interference of step length and velocity between FOG and non-FOG participants, we only found a trend towards significance between groups. This may be the result of having a large degree of variability in dual task interference and walking performance among the PD FOG participants. One potential explanation for this variability is that multiple strategies can be used by individuals during dual tasking which can influence spatiotemporal measures and the degree of dual task interference. Previous studies have described “posture first” and “posture second” strategies with the former being characterized by a degradation of cognitive performance to maintain gait and posture, and the latter being characterized by treating cognitive and gait tasks with an equal level of importance (Bloem et al., 2006). The “posture second” strategy appears to become increasingly prevalent in elderly PD patients which may lead to increased fall risk; however, this may vary depending on the individual’s perceived difficulty of a given cognitive task (Bloem et al., 2006).

There are a few limitations to consider when interpreting results of this study. First, due to technological limitations, the current study analyzed spatiotemporal metrics from the entirely of the time up and go trails rather than the walking and turning phases separately. Given the known contribution of turning to FOG behavior, future studies should consider investigating these phases separately. From a neuroimaging perspective scanning was performed at rest with the absence of a gait task due to the challenge of performing this in the scanner environment. Thus, it is difficult to determine whether the pattern of increased vermis connectivity would also be observed while FOG participants are actively engaged in a gait task. In addition, scanning was only performed while participants were on medication, which may have influenced the sensitivity of connectivity-behavioral associations for participants which only experience off-state FOG. Finally, this study did not compare functional connectivity to non-PD controls, limiting interpretability of whether the observed changes are disease specific. An expanded design of the fMRI component of this study in the future may help clarify interpretation.

Overall, this study demonstrates that increased vermal-cortical functional connectivity may contribute to impaired automaticity of gait in PD FOG. While the finding that increased functional connectivity is associated with dual task inference is correlational and does not provide causal evidence, future studies may be able to use this circuit model to experimentally modulate the cerebellum for FOG and impaired gait automaticity.

## Supplementary Information

Below is the link to the electronic supplementary material.

Supplementary file1 (DOCX 307 KB)

## Data Availability

The data that support the findings of this study are available upon reasonable request.
