# Supplementary figures and images for: Reduced automaticity in freezing of gait is associated with elevated cortico-cerebellar connectivity
Source: Brain Imaging Behav. 2025 Mar 18;19(3):637–46. doi: 10.1007/s11682-025-00996-w (PMC12198313; doi:10.1007/s11682-025-00996-w)

**Supplemental Figure 1. Vermis seed in MNI space**


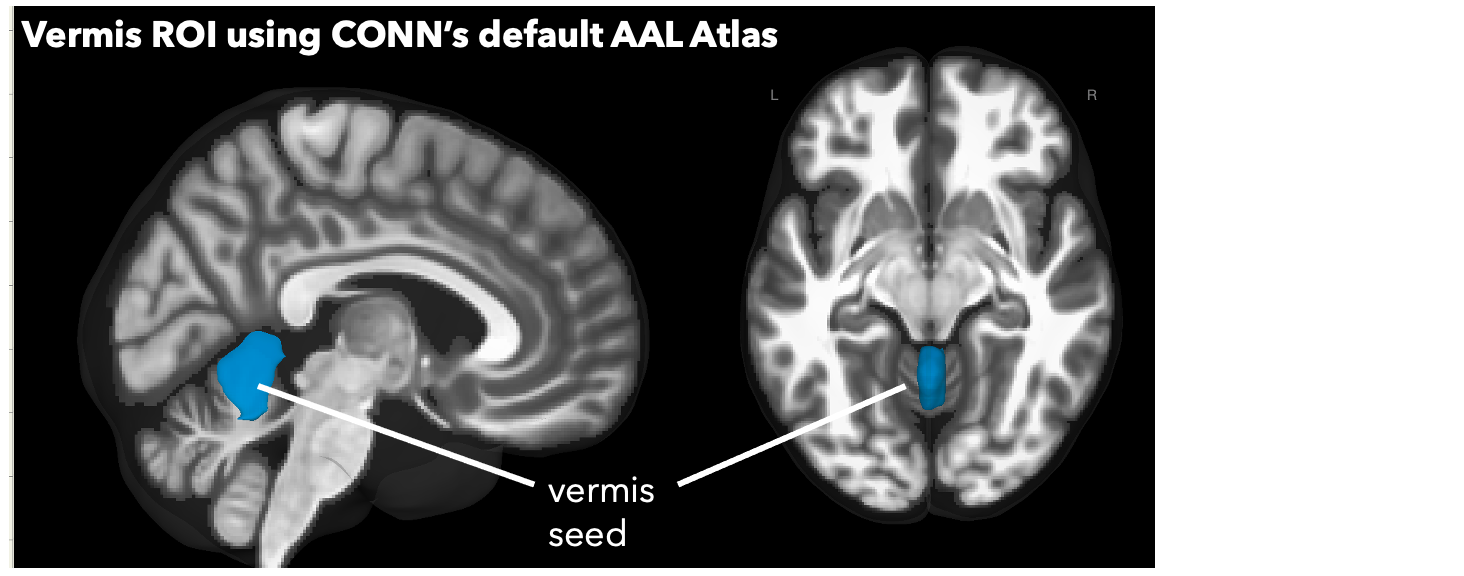

Supplement: Supplementary file 1 — Supplementary file1 (DOCX 307 KB) [file 11682_2025_996_MOESM1_ESM.docx]
